# Comprehensive Analysis Revealed the Potential Implications of m6A Regulators in Lung Adenocarcinoma

**DOI:** 10.3389/fmolb.2022.806780

**Published:** 2022-03-28

**Authors:** Lingling Xie, Rongyang Dai, Xudong Wang, Guangfei Xie, Zhihua Gao, Xinxin Xu

**Affiliations:** ^1^ Department of Laboratory Medicine, Affiliated Hospital of Nantong University, Nantong, China; ^2^ Department of Biochemistry and Molecular Biology, Southwest Medical University, Luzhou, China; ^3^ Department of Laboratory Medicine, People's Hospital of Sheyang County, Yancheng, China

**Keywords:** lung adenocarcinoma, m6A decoration, tumor microenvironment, prognosis, immunotargeted therapy

## Abstract

**Background:** The biological significance of RNA N6-methyladenosine (m6A) decoration in tumorigenicity and progression has been highlighted in recent studies, but whether m6A modification plays a potential role in tumor microenvironment (TME) formation and immune regulation in lung adenocarcinoma (LUAD) remains unclear.

**Methods:** m6A modification features were evaluated by analyzing the multi-omics features of 17 m6A regulators in over 1900 LUAD samples, and at the same time, the correlation between these modification patterns and TME characteristics was analyzed. An m6A score signature–based principal component analysis (PCA) algorithm was constructed to assess the prognosis and responses of individual patients to immunotherapeutic and targeted therapies.

**Results:** Three different m6A modification patterns were determined in 1901 LUAD samples, which were found to be related to diverse clinical outcomes *via* different biological pathways. Based on the m6A score extracted from the m6A-associated signature genes, LUAD patients were separated into high- and low-m6A score groups. It was discovered that patients with high m6A scores had longer survival, lower tumor mutation loads, and low PD-L1/PDCD1/CTLA4/TAG3 expression level. In addition, LUAD patients with high m6A scores displayed lower IC_50_ to some targeted drugs, including nilotinib, erlotinib, imatinib, and lapatinib.

**Conclusion:** m6A modification was significantly associated with the TME and clinical outcomes. These findings may help gain more insights into the role of m6A decoration in the molecular mechanism of LUAD, thus facilitating the development of more effective personalized treatment strategies.

## Introduction

Lung cancer is the most prevalent malignancy of all cancers, with top incidence and mortality rate in the world (Siegel et al., 2020). Based on the pathologic type, lung cancer can be separated into non–small cell lung cancer (NSCLC) and small cell lung cancer (SCLC). In addition, lung adenocarcinoma (LUAD) remains the most common subtype of NSCLC (Chen et al., 2019a). The development of LUAD is a long-term complicated process involving multiple steps, including interactions of multiple genes with external factors. Despite remarkable progress in the treatment of LUAD with respect to surgery, radiotherapy, chemotherapy, and immunotherapy, the prognosis of LUAD patients is still unsatisfactory due to undesirable response to treatment in addition to invasion and migration of tumor cells. Therefore, it is essential to further comprehend the underlying molecular mechanisms of the occurrence and progression of LUAD and find some clinically effective diagnostic and prognostic biomarkers for the sake of developing more promising individualized treatment strategies for LUAD.

N6 adenosine (m6A) methylation is extensively present in mRNAs, lncRNAs, and miRNAs. It is the most prevalent RNA modification type and plays an important role in various physiopathological processes (Zhao et al., 2017; Zaccara et al., 2019; Gu et al., 2020). m6A decoration is loaded with a reversible and dynamic process, which is regulated by various types of regulatory factors: the methyltransferases (writer), binding proteins (reader), and degrees (eraser) (Wang et al., 2020a). It is necessary to study these regulatory proteins and gain a better understanding about the mechanisms of m6A modification in gene regulation since these proteins have a great impact on m6A modification (Li et al., 2019a; Chen et al., 2020). Ample evidence has shown that abnormal expression and gene variation of m6A regulatory factors are related to the progression of malignant tumors and abnormal immune regulation (Chen et al., 2019b; Wang et al., 2020b; Shulman and Stern-Ginossar, 2020; Zhao et al., 2020). A comprehensive analysis of genetic variations and expression interference behind the heterogeneity of LUAD would promote the discovery of novel biomarkers and therapeutic targets based on m6A modification (Chen et al., 2019b; Li et al., 2019b; Wang et al., 2020b; Shulman and Stern-Ginossar, 2020; Zhao et al., 2020). Previous studies have reported abnormal expression patterns of m6A regulatory factors in LUAD (Liu et al., 2020a; Zhang et al., 2020a; Li et al., 2021a). Zhou et al. analyzed 21 potential m6A regulators involved in the tumor immune microenvironment of LUAD, based on which they constructed a risk signature and used it to define the tendency of immune cell infiltration in LUAD and predict the prognosis of LUAD patients. They found that m6A regulators played a critical role in the tumor immune microenvironment (TME) of LUAD (Zhou et al., 2021). Another study revealed that LUAD patients with high risk scores had poorer survival rates. The analysis of univariate and multivariate Cox regression showed that m6A-RPS was an independent prognostic risk element, and a significant association was observed between the expression of m6A-RPS and m6A modulators (Sun et al., 2021). More studies have demonstrated that m6A-related genes are efficacious biomarkers for early diagnosis, prognostic prediction, and efficacy evaluation of LUAD (Zhang et al., 2020a; Zhuang et al., 2020; Li et al., 2021a; Zhang et al., 2021). The m6A reader YTHDC2 suppressed LUAD tumorigenesis *in vivo* and *in vitro* by directly targeting the solute carrier 7A11 (SLC7A11) (Ma et al., 2021). In addition, LUAD tumor growth was effectively inhibited *via* targeting the METTL3-dependent m6A decoration of FBXW7 (Wu et al., 2021a).

Previous research studies have concentrated on single or a few m6A-related genes and included only a small sample size. However, the occurrence and progression of LUAD is a long-term gradually evolutionary process of numerous genes and multiple steps and methylation decoration patterns. The clinical significance of the tumor microenvironment (TME) landscape and m6A regulatory factors in LUAD remains largely unknown. The purpose of our present study was to systematically evaluate the multi-omics features of 17 m6A regulators and the m6A decoration patterns by integrating the genomic and transcriptomic data of more than 1900 LUAD samples to see whether the m6A decorative pattern was associated with the TME characteristics. In addition, based on m6A regulators and related genes, we built a score system for quantifying m6A decoration patterns in individual tumors and forecasting the clinical response of LUAD patients to immune-targeted therapies. Moreover, we developed a nomogram based on the scoring system (m6Ascore signature) and some important clinical trials to predict the prognosis of LUAD patients. The results obtained in our study suggested that m6A modification played a pivotal role in forming various TME profiles which could serve as a guide for planning therapeutic intervention schemes for LUAD.

## Methods

### Collection and Pretreatment of Publicly Available Expression Data Sets

Clinical features of LUAD and gene expression data were collected retrospectively from TCGA (https://cancergenome.nih.gov/) and NCBI GEO databases (https://www.ncbi.nlm.nih.gov/geo/). Twenty-three m6A regulatory factors were obtained (Supplementary Table S1) (Chen et al., 2019b; Li et al., 2021b; Zhou et al., 2020; Liu et al., 2020b; Arguello et al., 2017; Warda et al., 2017; Barros-Silva et al., 2020; Wu et al., 2021b). TCGA somatic mutation data had been captured through the utilization of TCGAbiolinks R package (Colaprico et al., 2016) and visualized through application of the maftools R package (Mayakonda et al., 2018). The copy number variation (CNV) datasets were obtained from the Xena Public Data Center (https://xenabrowser.net). The copy number variation landscape of these 23 m6A regulators in human chromosomes was identified using R package “Rcircos”. TCGA–LUAD RNA-seq data (FPKM format) were downloaded from the Genomic Data Commons (GDC, https://portal.gdc.cancer.gov/) and converted into transcripts per kilobase million (TPM) format. The Gene Expression Omnibus (GEO) database was used to make a comprehensive query on all qualified LUAD data sets. A total of seven datasets [GSE30219 (Xie et al., 2011), GSE30219 (Rousseaux et al., 2013), GSE31210 (Okayama et al., 2012), GSE37745 (Botling et al., 2013), GSE50081 (Der et al., 2014), GSE68465 (Shedden et al., 2008), and GSE72094 (Schabath et al., 2016)] were obtained to represent different LUAD-independent studies. All datasets contained clinical data and survival information. The platform files and survival data of these datasets are shown in Supplementary Table S2. In accordance with the corresponding annotation file, the probe was transformed into a gene symbol. For genes with set signals of multiple probes, their values were averaged to produce a single gene expression value. At last, they were further combined to a meta-queue through the “ComBat” algorithm using the “sva” package (Leek et al., 2012) to decrease the batch effect from abiotic deviations.

### Unsupervised Clustering for 17 m6A Regulators

By integrating the eight datasets, 17 out of the 23 m6A modulators were used to identify different m6A decorative patterns mediated by the m6A modulators. They included five writers (RBM15, METTL3, WTAP, RBM15B, and ZC3H13), one eraser (FTO), and 11 readers (YTHDC2, YTHDC1, YTHDF2, YTHDF1, YTHDF3, IGF2BP2, IGF2BP3, HNRNPC, HNRNPA2B1, LRPPRC, and FMR1). Based on the expressions of the 17 m6A regulatory factors, we performed an unsupervised cluster analysis to discover different m6A decoration patterns, and the patients were categorized for subsequent analysis. The abovementioned steps were performed by applying the “ConsensusClusterPlus” software package (Wilkerson and Hayes, 2010) and repeated 1000 times to ensure stability of the classification.

### Gene Set Variation Analysis and Functional Annotation

To investigate the discrepancy in biological processes between the m6A decorative patterns, GSVA enrichment analysis was performed by using “GSVA” R package knowing that GSVA is an unsupervised and nonparametric method usually used to appraise changes in pathway and biological process activities in expression datasets (Hänzelmann et al., 2013). The gene sets of “c2.cp.kegg.v7.2.symbols” were downloaded from MSigDB (http://www.gsea-msigdb.org/gsea/msigdb) to be used for analysis of GSVA. After adjustment, *p* < 0.05 indicated statistical significance. Functional annotation of m6A-associated genes was analyzed by using the “clusterProfiler” R package (Wu et al., 2021c), and the cutoff value of the FDR was <0.05.

### Estimation of the TME

The level of infiltration of diverse immune cells, including the TME and adaptation of innate immune cell types, was quantified by single sample GSEA (ssGSEA) (Barbie et al., 2009; Charoentong et al., 2017). Immune cell and stromal cell score in malignant tumors were estimated by the expression data (estimation) algorithm (Becht et al., 2016) using the distinct properties of the transcription profile to deduce the nature of the tumor cell and purity of the tumor. The infiltration level of stromal and immune cells was predicted by the score of stromal and immune cells calculated by the ESTIMATE algorithm and used as the basis for inferring tumor purity. LUAD tissue with enriched infiltration of immune cells indicated a better immune score and poorer tumor purity.

### Identification of Differentially Expressed Genes Between Distinct m6A Phenotypes

To find m6A-associated genes, patients were categorized into three different m6A decoration patterns according to the expression of the 17 m6A regulatory factors. The deg between different decorative patterns was demonstrated by the empirical Bayesian approach of limma R package (Ritchie et al., 2015) *p* value <0.001 was defined as the significant standard to determine DEGs.

### Construction of the m6A-Related Gene Signature (m6A score)

To determine the m6A decoration pattern of individual tumors, we constructed a scoring system to assess the m6A decoration pattern of individual LUAD patients. The genetic marker associated with m6A is called m6Ascore. Specifically, overlapping DEGs discovered from different m6A clusters were standardized and extracted to facilitate analyzing the prognosis of each gene using the univariate Cox regression model. Genes with vital prognosis were collected for principal component analysis (PCA) to construct m6A-associated gene markers. Principal components 1 and 2 were regarded as signature fractions. The merit of this approach is that the score is focused on the largest correlated (or anti-correlated) gene block in the set, while the contribution of genes that are not interacted with other members of the set is underweighted. Then, we use a similar formula from previous research to determine m6Ascore (Zhang et al., 2020b):

m6Ascore=∑(PC1i+PC2i),

where i is the expression of terminally determined genes associated with the m6A phenotype.

### Gene Set Enrichment Analysis and Functional Annotation

Using the clusterProfiler R software package, GSEA was executed to deduce biological processes associated with m6A regulators, and we regarded *p* value <0.05 as statistically significant.

### Estimation of Drug Sensitivity

Drug sensitivity was evaluated by using the pRRophetic R software package (Geeleher et al., 2014a) and defined by the concentration essential for 50% inhibition of cell growth (IC_50_) (Geeleher et al., 2014b).

### Statistical Analysis

**FIGURE 1:**
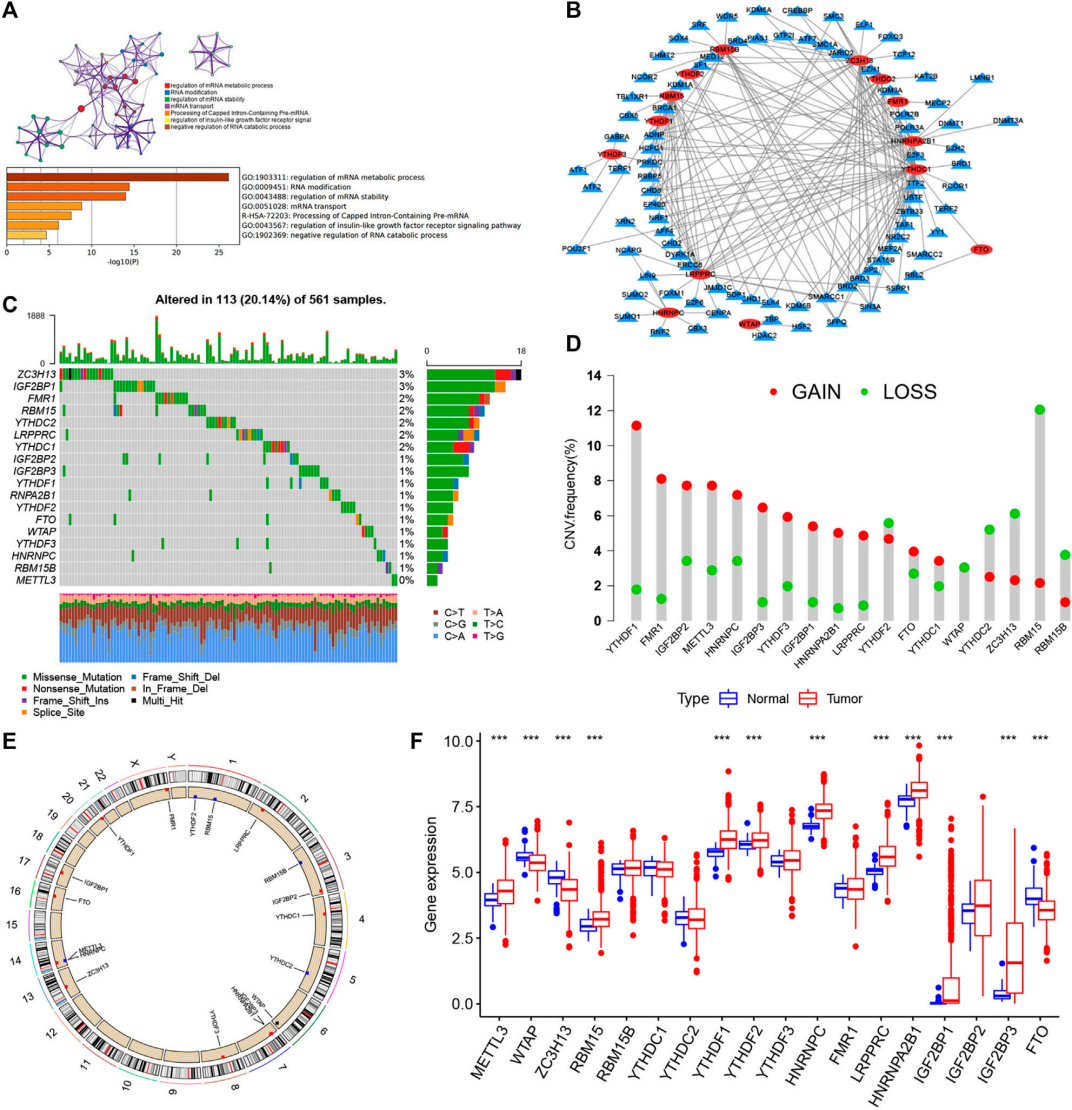
Landscape of genetic and expression variation of m6A regulators in LUAD. **(A)** Metascape enrichment network and heat map of enrichment items in the input gene list colored with *p* value. **(B)** Determination of transcription factors related to 23 m6A regulatory factors by Pearson’s correlation analysis (|Cor| > 0.5 and *p* < 0.001). **(C)** Mutation frequency of 23 m6A regulatory factors in 561 LUAD patients in TCGA–LUAD cohort. Each column represents an individual patient. The abovementioned barplot is TMB, and the number on the right represents the mutation frequency of each regulatory factor. The barplot on the right shows the proportion of each variation type. The stacked barplot below shows the transformation section in each example. **(D)** CNV variation frequency of m6A regulatory factors in TCGA–LUAD cohort. The height of the column represents the frequency of change. Delete frequency, green dot; amplified frequency, red dot. **(E)** Location of CNV change of m6A regulatory genes on 23 chromosomes by TCGA–LUAD cohort. **(F)** Expression of 23 m6A regulatory factors in normal and LUAD tissues. Tumors, red; normal, blue. The upper and lower ends of the box represent the quartile range of values. The lines in the box represent intermediate values, and the real points represent outliers. The asterisk represents the statistical *p* value. (*p < 0.05; **p < 0.01; ***p < 0.001).

The limma R package was used for DEG expression analysis, and Spearman’s method was used for correlation analysis. The statistical difference between two groups was calculated by using the Wilcoxon rank sum test. The Kruskal–Wallis test was used for comparing more than two groups. The univariate Cox regression model was used to estimate the risk ratio (HR) of m6A regulatory factors. The multivariate Cox regression model was used to determine the independent prognostic factors. The correlation between the m6A decoration pattern and prognosis with the“survminer” package in R was analyzed by the Cox proportional hazard model and Kaplan–Meier survival analysis. Using surv-cutpoint function from the “survival” software package, samples were stratified into high- and low-m6Ascore groups. The mutation landscape of m6Ascore patients in the TCGA–LUAD cohort with high or low subgroups was presented by the waterfall function of the maftools software package. Patients with complete clinical data were enrolled in ultimate multivariate Cox analysis. The results were visualized using the “forestplot” software package in R. The receiver operating characteristic (ROC) curve was used to assess the prognosis classified performance and other clinical factors of m6Ascore, and the area under the curve (AUC) was determined by using the “survivalROC” package. Using the clinical characteristics and m6Ascore signature as input, multivariable Cox regression analysis and variable selection were used to determine the strong combination of these predictors. Then, we built up a quantitative line chart with the “rms” package in R to predict the individual three- and Figure 1A five-year survival probabilities. To assess the prediction performance of the nomogram, we used the bootstraps method to calculate the C-index. At the same time, the calibration curves, with the Hosmer–Lemeshow test, were also used to judge the consistency between the model prediction values and actual results. All statistical *p* values were two-tailed, and *p* < 0.05 was considered as statistical significant. The data processing was accomplished in R 3.6.2 software.

## Results

### Genetic Variation Landscape of m6A Regulators in LUAD

Twenty-three m6A regulators including two erasers, eight writers, and 13 readers (“erasers”: ALKBH5 and FTO; “writers”: METTL14, METTL3, METTL16, WTAP, VIRMA, ZC3H13, RBM15B, RBM15; and “readers”: YTHDC1, YTHDF1, YTHDC2, YTHDF2, FMR1, YTHDF3, HNRNPC, LRPPRC, RBMX, IGF2BP1, HNRNPA2B1, IGF2BP2, and IGF2BP3) were initially obtained, and by integrating multiple datasets, we finally included 17 m6A regulators including five writers (METTL3, RBM15, RBM15B, WTAP, and ZC3H13), one eraser (FTO), and 11 readers (YTHDC1, YTHDC2, YTHDF1, YTHDF2, YTHDF3, IGF2BP2, IGF2BP3, HNRNPA2B1, HNRNPC, FMR1, and LRPPRC) in this study. GO enrichment and Metascape analyses were performed on the 17 m6A regulatory factors, and the biological processes of valuable enrichment are concluded in Figure 1A. The transcription factor (TF) subset was from Cistrome (http://cistrome.org/). We, thus, obtained the corresponding TF expression values in TCGA database. Through Pearson’s correlation analysis (|Cor| > 0.5 and *p* < 0.001), we identified the TFs related to the 17 m6A regulators (Supplementary Table S3). As indicated in Figure 1B, most TFs were involved in the expression of YTHDC1 (n = 41), followed by ZC3H13 (n = 27). Moreover, we concluded the incidence of CNVs and somatic cell mutations of the 17 m6A regulators in LUAD. m6A regulator mutation was observed in 113 (20.14%) of the 561 samples, with the mutation frequency of ZC3H13 being the highest, followed by FMR1, RBM15, YTHDC2, LRPPRC, and YTHDC1, although METTL3 showed no mutations in LUAD samples (Figure 1C). Taking the relatively high mutation frequency of the “writer” gene ZC3H13 into account, further exploration suggested that mutations in ZC3H13 did not affect the expression of other m6A regulators (Supplementary Figure S1). The subsequent analysis of 17 m6A regulators demonstrated that mutations in CNV are widespread. YTHDF1, FMR1, IGF2BP2, METTL3, HNRNPC, IGF2BP3, YTHDF3, IGF2BP1, HNRNPA2B1, LRPPRC, YTHDC1, and FTO extensive CNV amplification was shown. In sharp contrast to this, YTHDF2, WTAP, YTHDC2, ZC3H13, RBM15, and RBM15B displayed general CNV deficiency (Figure 1D). Figure 1E showed the CNV change positions of the 17 m6A regulatory genes on chromosomes. To determine if the aforementioned genetic variants affected the m6A regulatory factor expression in LUAD patients, a difference in the mRNA expression of regulators between normal and LUAD samples was examined in TCGA–LUAD cohort (Figure 1F), and it was found that CNV changes were probably the primary factor interfering with the expression of m6A regulators. To identify the correlation between these m6A regulatory factors and prognosis of LUAD patients, we used Cox regression analysis. The results revealed that METTL3, YTHDC1, YTHDC2, and FTO were protective factors significantly correlated with longer overall survival rates, while WTAP, RBM15, HNRNPC, LRPPRC, IGF2BP2, and IGF2BP3 were risk factors (Supplementary Figure S2A). Of them, METTL3 and HNRNPC were independent prognostic genes for LUAD (Supplementary Figure S2B). The abovementioned analysis revealed a highly heterogenous pattern of altered genetics and expression in the m6A regulators among normal and LUAD samples, suggesting that imbalance in the expression of m6A regulators plays a key role in the development and progression of LUAD.

### m6A Methylation Decoration Patterns Mediated by 17 Regulators

**FIGURE 2:**
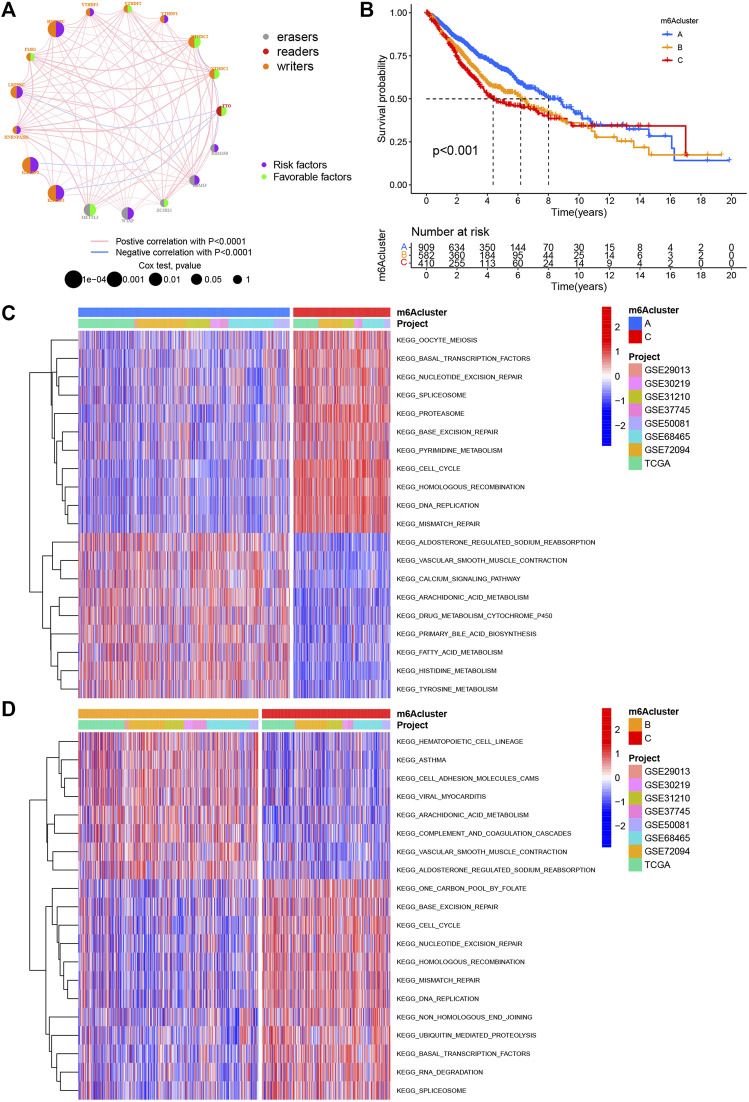
m6A methylation modification pattern and biological characteristics of each pattern. **(A)** Interaction of m6A regulators in LUAD. The circle size represents the impact of each regulatory factor on prognosis, and the range of calculated value by log-rank test was *p* < 0.0001, *p* < 0.001, *p* < 0.01, *p* < 0.05, and *p* < 1. Left half of the circle: purple represents prognostic risk factors and green represents prognostic favorable factors. Right half of the circle: the types of m6A regulators. The line connecting regulatory factors represents the interaction between regulatory factors, and the thickness represents the correlation intensity between regulatory factors, showing a negative association with blue and a positive association with pink. **(B)** Survival analysis based on three m6A patterns in 1901 patients from seven GEO cohorts (GSE30219, GSE31210, GSE29013, GSE37745, GSE68465, GSE50081, and GSE72094) and one TCGA–LUAD cohort, including 909 cases in m6Acluster A, 582 in m6Acluster B, and 410 in m6Acluster C. Kaplan–Meier curves with log-rank *p* value less than 0.001 show significant survival differences in survival rates between the three m6A modification modes. The overall survival of m6Acluster B was significantly better than that of the other two m6Aclusters. **(C,D)** Activation state of biological pathways in different m6A modification patterns as shown by GSVA enrichment analysis. The biological processes were visualized by using heat maps, with red representing activated pathways and blue representing inhibited pathways. LUAD queue was used as sample annotations. C: m6Acluster C vs. m6Acluster A; D: m6Acluster C vs. m6Acluster B.

**FIGURE 3:**
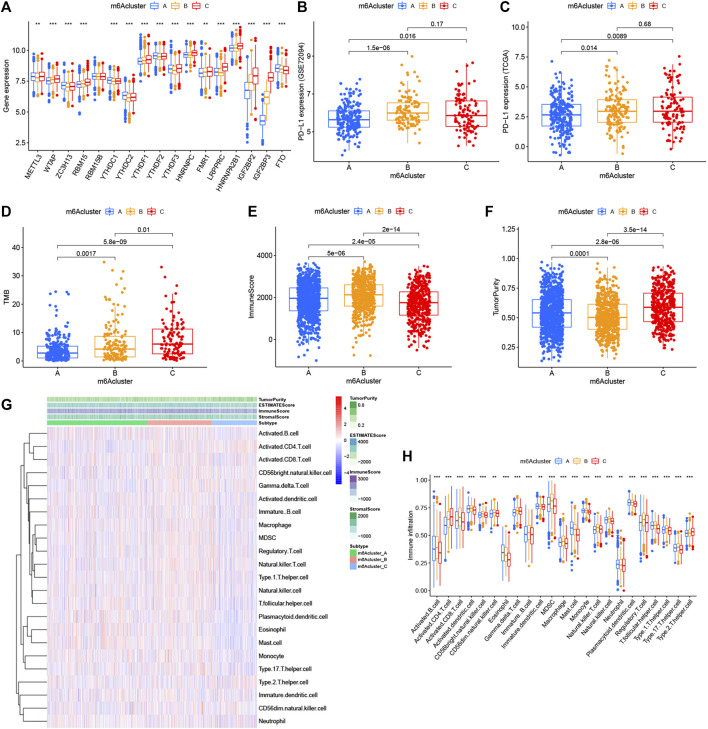
Relationship between the three m6A modification patterns and expression of m6A regulators and TME landscape. **(A)** Expression difference of 17 m6A regulators in the three m6A modification patterns. m6Acluster-A, blue; m6Acluster-B, yellow; m6Acluster-C, red. The top and bottom of the box represented the range of quartiles of values. The lines in the boxes represented median values, and the solid dots represented outliers. The asterisk indicated the statistical *p* value (*p < 0.05; **p < 0.01; ***p < 0.001). **(B)** Expression difference of PD-L1 (CD274) in the three m6A modification patterns in the GSE72094 cohort. **(C)** Expression difference of PD-L1 (CD274) in the three m6A modification patterns in TCGA cohort. **(D)** Difference of TMB in the three m6A modification patterns in TCGA–LUAD cohort. **(E)** Comparison of immune scores across the three m6A modification patterns. **(F)** Comparison of tumor purity across the three m6A patterns. **(G)** TME landscape of the three m6A modification patterns. Tumor purity, immune score, ESTIMATE score, stroma score, and m6A clustering were noted above. Red indicated a high degree of infiltration of TME infiltrating cells, and blue indicated a low degree of infiltration of TME infiltrating cells. **(H)** Abundance of each TME infiltrating cell in the three m6A decoration modes. The upper and lower ends of the box represented the quartile range of values. The lines in the box represented intermediate values, and the real points represented outliers. The asterisk represented the statistical *p* value. (***p < 0.001; **p < 0.01; *p < 0.05). m6Acluster-A, blue; m6Acluster-B, yellow; m6Acluster-C, red. For comparisons of the three groups, the Kruskal–Wallis test was used.

Eight data sets containing operating system data and clinical information (TCGA–LUAD, GSE29013, GSE30219, GSE31210, GSE37745, GSE50081, GSE68465, and GSE72094) were included into one meta-cohort. To learn the comprehensive landscape of m6A regulator interactions, the m6A regulatory network was used to define the prognostic significance of connections between the m6A regulators in LUAD patients (Figure 2A). The results showed that the crosstalk between the regulators, readers, and erasers played a key role in the formation of various m6A decorative patterns and were related to the occurrence and development of cancer. Based on these findings, we used the “ConsensusClusterPlus” package in R to categorize the patients qualitatively into different m6A decoration modes according to the expression of the 17 m6A regulators. Finally, three different decoration modes were determined based on unsupervised clustering. We named them as m6A cluster A (n = 909), B (n = 582), and C (n = 410) (Figure 2B). Among them, the m6A cluster-A showed an obvious survival advantage, while m6A cluster-C showed the worst prognosis (Figure 2B). In addition, we also noticed that the expression levels of these m6A regulators differed significantly between the different m6A decoration patterns. WTAP, RBM15, HNRNPC, LRPPRC, IGF2BP2, and IGF2BP3 were elevated markedly in the m6A cluster-C subtype, while YTHDC1, YTHDC2, and FTO were increased in the m6A cluster-A subtype (Figure 3A).

### TME Landscape in Distinct m6A Decoration Patterns

With the aim of investigating the biological behavior between these different m6A decoration patterns, we executed an analysis of GSVA enrichment and found that m6A cluster-A exhibited enrichment pathways related to complete immune activation, which included drug metabolism cytochrome P450, fatty acid metabolism, tyrosine metabolism, and histidine metabolism (Figures 2C,D **).** Knowing that tumor mutation burden (TMB) and PD-L1 are mature biomarkers for predicting anti–PD-1/L1 treatment response, we compared the TMB and expression level of PD-L1 in the three different m6A decoration clusters, finding that PD-L1 (Figures 3B,C) and TMB (Figure 3D) were obviously downregulated in the m6A cluster-A subtype. In addition, we quantified the overall infiltration (immune score) and tumor cell purity (tumor purity) of immune cells under the three decoration modes using the ESTIMATE algorithm and found that m6Acluster-A and -B had the higher immune score than m6Acluster-C (Figure 3E). Conversely, m6A cluster-C had higher tumor purity than m6A cluster-A and m6A cluster-B (Figure 3F). Subsequently, we performed a ssGSEA analysis to explore the relative abundances of 23 immune infiltration cells in the three subtypes (Figures 3G,H). Antitumor lymphocyte cell sub-populations, such as activated CD8+ T cells, NK cells, and NK T cells, were mainly concentrated in the m6A cluster-B subtype. Among them, CD56 dim natural killer cell, immature B cell, MDSC, macrophage, monocyte, regulatory T cell, type 1 T helper cell, and type 17 T-helper cells were also significantly infiltrated in m6A cluster-B tumors. These results suggested that m6A cluster-B tumors were embraced by more nontumor components, such as stromal and immune cells.

### m6A Phenotype–Associated DEGs in LUAD

**FIGURE 4:**
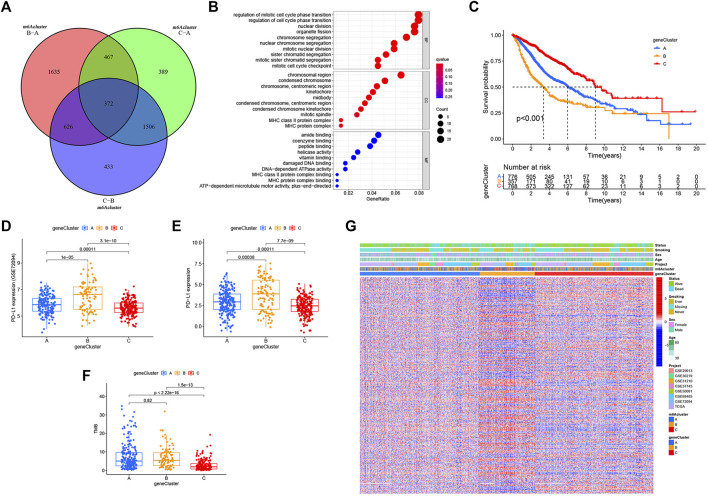
m6A phenotype–related DEGs and functional annotation. **(A)** Venn diagram of 372 m6A-related DEGs between the three m6A decoration patterns. **(B)** Functional annotation for phenotype-related DEGs associated with the prognosis using GO enrichment analysis. **(C)** Estimation of the survival curve of m6A phenotype–related gene markers by Kaplan–Meier plotter (*p* < 0.001, log-rank test). **(D)** Expression difference of PD-L1 (CD274) in the three geneclusters in the GSE72094 cohort. **(E)** Expression difference of PD-L1 (CD274) in the three gene Clusters in TCGA cohort. **(F)** Difference in TMB between the three gene clusters in TCGA–LUAD cohort. **(G)** Overlapping m6A phenotype–related DEGs associated with prognosis as unsupervised clustering, and the patients were divided into different genomic subtypes defined as gene clustering A–C. Gene signature subtypes, m6A clusters, items, age, gender, smoking status, and life status were used as patient annotations.

Despite the classification of LUAD patients into three m6A-modified phenotypes based on a consensus clustering algorithm for m6A regulator expression, the underlying genetic changes and expression disorders in these phenotypes remained unclear. Therefore, we further observed changes in the potential transcriptional expression of the three m6A decoration patterns in LUAD. 372 overlapping DEGs between the three m6A decoration patterns were determined by the empirical Bayesian method (Figure 4A). Next, univariate Cox analysis was executed for these 372 DEGs to determine the genes associated with prognosis. Finally, 303 m6A phenotype–related DEGs associated with prognosis were regarded as m6A-associated signature genes (Supplementary Table S4). The GO analysis of the enrichment for these characteristic genes showed significant changes in biological processes associated with cell cycle (Figure 4B and Supplementary Table S5). Based on the 303 most representative genes associated with the m6A phenotype, we executed unsupervised consensus cluster analysis and acquired three stable transcriptome phenotypes (m6A geneCluster-A, m6A geneCluster-B, and m6A geneCluster-C) (Supplementary Figure.S2E). The results showed that patients in geneCluster-B displayed the worst prognosis (Figure 4C) and the highest PD-L1 expression (Figures 4D,E) and TMB (Figure 4F). As shown in Figures 4D–F, the patients in geneCluster-C had the lowest PD-L1 expression and TMB but the longest overall survival. Moreover, surprisingly, the prognostic risk genes (RBM15, HNRNPC, LRPPRC, IGF2BP2, and IGF2BP3) were all poorly expressed in geneCluster-C, and the prognostic-friendly genes (YTHDC1, FTO, and YTHDC2) were highly expressed (Supplementary Figure S2G). Similarly, the prognostic risk gene (WTAP) was highly expressed in geneCluster-B, while the prognostic-friendly gene (METTL3) was less expressed (Supplementary Figure S2G). The prognostic differences between geneCluster-A, B, and C and the corresponding expressions of YTHDC1, YTHDC2, RBM15, HNRNPC, LRPPRC, IGF2BP2, IGF2BP3, METTL3, and WTAP were consistent with the results of Cox regression analysis (Supplementary Figures S2A,B). All these results indicated that the three stable transcriptomic phenotypes (geneCluster-A, B, and C) could well-distinguish between different prognostic populations.

### Construction of the m6Ascore Signature and Exploration of its Clinical Relevance

**FIGURE 5:**
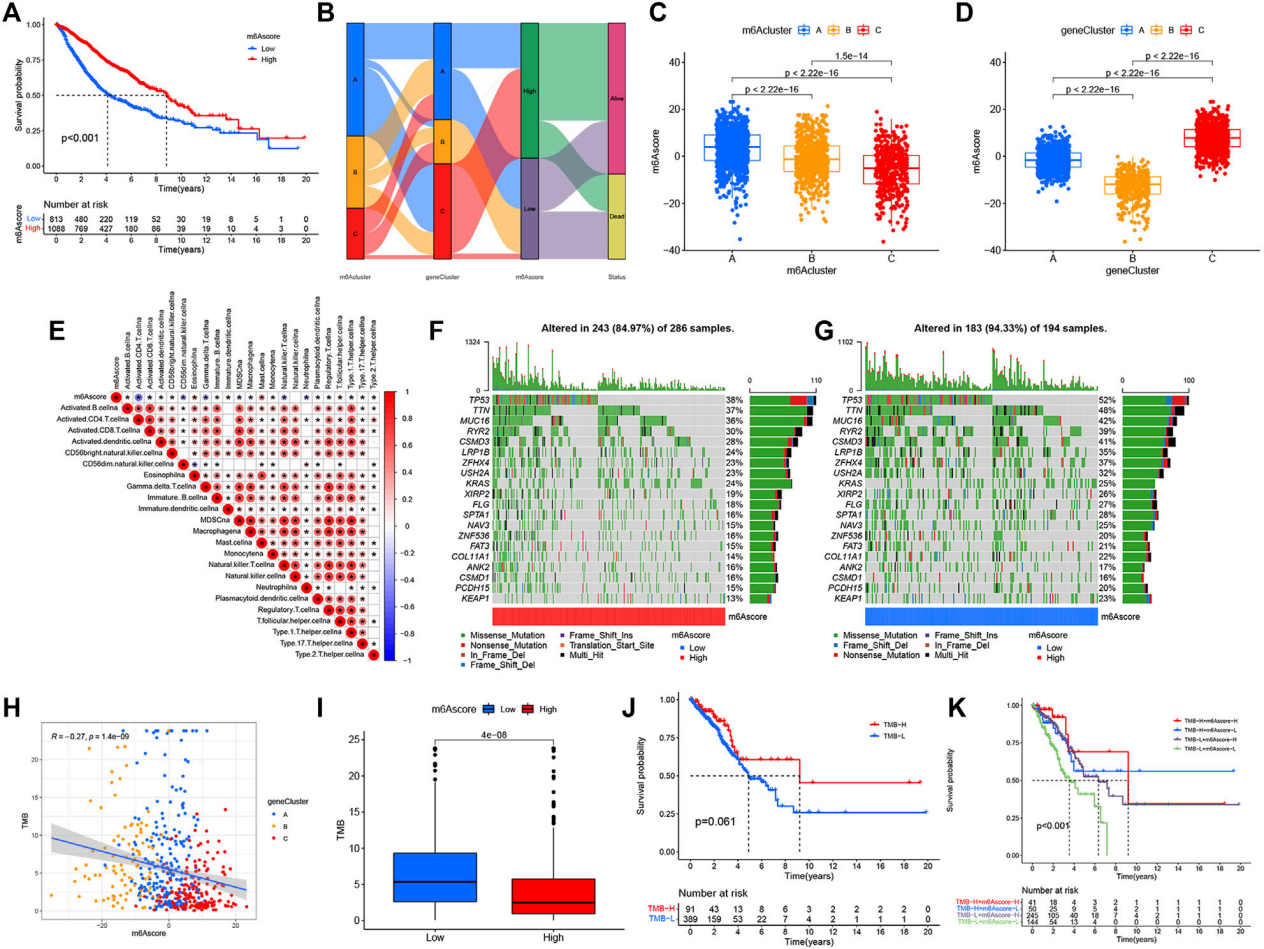
Construction of the m6Ascore signature and tumor somatic mutation. **(A)** Survival analysis of patients with low m6Ascore (813 cases) and high m6Ascore (1088 cases) by Kaplan–Meier curve (*p* < 0.001, log-rank test). **(B)** Alluvial map showing changes in m6Aclusters, geneCluster, m6Ascore, and status. **(C)** Comparison of m6Ascore across the three m6A modification patterns. **(D)** Comparison of m6Ascore across three geneClusters. **(E)** Spearman’s analysis of correlations between m6Ascore and TME infiltrating cells. Blue represented a negative association and red represented a positive association. **(F,G)** Waterfall diagram of tumor somatic mutation established by high m6Ascore **(F)** and low m6Ascore **(G)**. Each column indicated an individual patient. The figure above showed TMB, and the number on the right represented the mutation frequency of each gene. The bar graph on the right showed the proportion of each variation type. **(H)** Correlations between TMB, m6Ascore, and geneCluster using Spearman’s analysis. **(I)** Comparison of TMB between high- and low-m6Ascore groups. **(J)** Kaplan–Meier curves for survival analysis of patients with low (389) and high (91) TMB in TCGA–LUAD cohort (*p* = 0.061, log-rank test). **(K)** Kaplan–Meier curve analysis of survival in “TMB-H + m6Ascore-H”, “TMB-H + m6Ascore-L”, “TMB-L + m6Ascore-H”, and “TMB-L + m6Ascore-L” groups in TCGA–LUAD cohort (*p* < 0.001, Log-rank test). TMB-H, high TMB; TMB-L, low TMB; m6Ascore-H, high m6Ascore; m6Ascore-L, low m6Ascore.

While we discovered that m6A modifications could be critical in regulating prognosis and immune infiltration, correlation analysis was conducted only on the basis of patient groups and was unable to accurately forecast the m6A methylation decoration pattern in a single tumor. Considering the individual heterogeneity and complexity of m6A decoration, we constructed a score system to quantify the m6A modification patterns of individual LUAD patients according to these phenotypically associated genes, which we called m6Ascore. Next, we tried to further define the value of m6Ascore in forecasting the prognosis of LUAD patients. We determined the critical value −1.244678 by the measurement package in R and separated the patients into a low-m6Ascore group and a high-m6Ascore group. Patients in the high-m6Ascore group showed significant survival superiority. The median OS was twice as long as that of patients in the high-m6Ascore group (8.4 vs. 4.1) (Figure 5A). We expressed the attribute changes of individual patients in an alluvial map **(** Figure 5B). Our subgroup analysis on different groups by age, sex, stage, smoking status, and EGFR/Kras/TP53/STK11 mutation status showed that the prognosis of patients with high m6Ascore was significantly better than that of patients with low m6Ascore (age ≤ 65 subset, age > 65 subset, female subset, male subset, ever smoking subset, never smoking subset, stage I/II subset, stage III/IV stage, EGFR wt subset, STK11 wt subset, Kras wt/mut subset, and TP53 wt/mut subset), which further showed that the predictive performance of m6Ascore was reliable in various subgroups of LUAD patients (Supplementary Figure S3). Subsequently, a further comparison of m6Ascore differences between different subgroups revealed that these subsets (female subset, never smoking subset, EGFR-Mut subset, Kras-WT subset, TP53-WT subset, and alive subset) had higher m6Ascore (Supplementary Figure S4). Given that m6Ascore could well-predict patient prognosis, we further compared m6Ascores between distinct m6A modification patterns. m6Acluster-A was found to have the highest m6Ascore, followed by m6Acluster-B and m6Acluster-C with the lowest m6Ascore (Figure 5C). This simply demonstrated the accuracy of m6A modification patterns in distinguishing various prognostic patients (Figure 2B). Similarly, Figure 5D also demonstrated the accuracy of the geneCluster in the classification of patients with different prognoses (Figure 4C). Furthermore, Spearman’s analysis was used to detect the relationship between the relative abundance of 23 immune infiltrating cells and m6Ascore. The correlation matrix heat map showed a significantly negative correlation of m6Ascore with activated CD4 T cells and a significantly positive connection with mast cells (Figure 5E).

### The Association Between m6Ascore and TME Cell Infiltration and Functional Annotation

ssGSEA analysis showed that low-m6Ascore tumors were significantly infiltrated by CD4 T, CD8 T, activated B, CD56 bright NK cell, gamma delta T cell, MDSC, macrophage, immature B cell, NK cell, regulatory T cell, and type 1 T helper cell, while high-m6Ascore tumors were significantly infiltrated by eosinophil, mast cell, monocyte, plasmacytoid dendritic cell, and T follicular helper cell (Supplementary Figure S5A). The ESTIMATE algorithm was used to quantify the tumor purity and immune score of each sample. The Wilcoxon rank sum test revealed significant differences in immune score and tumor purity between high- and low-m6Ascore groups, indicating that tumor purity was higher (Supplementary Figure S5C) and the immune score was lower (Supplementary Figure S5B) in the high-m6Ascore group than the low-m6Ascore group. The GSEA analysis of the two groups showed that DNA replication, p53 signaling pathway, cell cycle, IL-17 signaling pathway, and progesterone-mediated oocyte maturation were markedly enriched in low-m6Ascore tumors (Supplementary Figure S5D). GSVA enrichment analysis was then conducted to investigate the biological behavior between m6Ascore groups.

### Tumor Somatic Mutation and the Role of m6Ascore in Predicting Immunotherapeutic and Targeted Therapy Benefits

**FIGURE 6:**
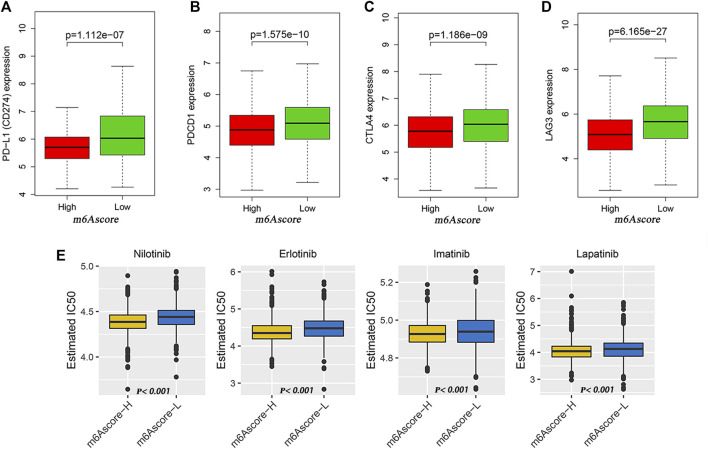
Potential of m6Ascore in predicting therapeutic response. **(A–D)** Wilcoxon rank sum test shows significant differences in the expression of PD-L1 **(A)**, PDCD1 **(B)**, CTLA4 **(C)** and LAG3 **(D)** between low- and high-m6Ascore groups. **(E)** Comparison of drug sensitivity between high- and low-m6Ascore groups. Distribution of IC_50_ of nilotinib, imatinib, erlotinib, and lapatinib between high- and low-m6Ascore groups.

Then, we analyzed the differences of somatic mutation distributions in TCGA–LUAD cohort between high- and low-m6Ascore groups by maftools R package, and we found that the tumor mutation burden was more extensive in the low-m6Ascore group than in the high-m6Ascore group. The mutation rate of the fifth most significant mutation gene was 28% in the high-m6Ascore group vs. 41% in the low-m6Ascore group (Figures 5F,G **).** Quantitative analysis of TMB demonstrated that tumors with low m6Ascore were significantly associated with high TMB (Figure 5I), showing a significant negative correlation between m6Ascore and TMB (Figure 5H). We further explored the correlation between TMB and m6Ascore and the prognosis of patients and found that low TMB seemed to have a worse prognosis (Figure 5J). Notably, when TMB and m6Ascore were both low, the prognosis of the patients was the worst (Figure 5K). These findings may help better understand the impact of m6Ascore classification on genomic variation and revealed the potential complex interactions between single somatic mutations, m6A decoration, and patient outcomes. The Wilcoxon rank sum test showed that the expression of immune checkpoints was obviously different (PD-L1, PDCD1, CTLA4, LAG3) between the high- and low-m6Ascore groups. The low-m6Ascore group showed higher immune checkpoint expression (Figures 6A–D). Ample evidence suggested that patients at a high TMB status and high immune checkpoint expression exhibited sustained clinical reactions to anti–PD-1/PD-L1 immunotherapy. Hence, the results mentioned above may not only indirectly prove that variations in the tumor m6A modification mode are key factors mediating the clinical response to anti–PD-1/PD-L1 immunotherapy but also confirm the value of m6Ascore in forecasting the outcome of immunotherapy. As targeted therapy has been widely used in LUAD therapy, it is important to identify subgroups of patients who may be more sensitive to some drugs. Here, we predicted the response of patients in the high- and low-m6Ascore groups to several commonly used drugs. As shown in Figure 6E, patients with high m6Ascore were more sensitive to nilotinib, erlotinib, imatinib, and lapatinib in the LUAD cohort, which may provide useful evidence for guiding personalized treatment strategies for LUAD patients.

### Construction and Evaluation of the Nomogram

In view of the value of m6Ascore in judging the prognosis of patients, we further used the Cox proportional hazard model to explore the independent prognostic factors of LUAD patients. As shown in Supplementary Figures S6A,B, age, smoking status, stage, and m6Ascore were all independent prognostic factors. Based on these findings, we constructed a nomogram to verify the role of various factors in forecasting the prognosis of patients (Supplementary Figure S6C). Based on the results of multivariate Cox analysis, the score was assigned to each factor in the rosette, and the total rosette score was obtained from the sum of the individual scores of all predictors. Using the total score, the 3- and 5-year survival rates of patients could be estimated by predicting the total score downward. Compared with the ideal model, the 3- and 5-year calibration curves also revealed good consistency, which further indicated that the nomogram was stable in predicting outcomes in patients with LUAD (Supplementary Figure S6D).

## Discussion

As a dynamic and reversible process regulated by m6A regulatory factors, m6A modification promotes or suppresses malignant behavior mainly by modulating the expression of targeted oncogenes or oncogenes (Li et al., 2017; Ma et al., 2017; Bai et al., 2019). Growing evidence has clarified that m6A modification plays critically important roles in innate immune, inflammatory, and antitumor effects through interplay with different m6A regulators (Shulman and Stern-Ginossar, 2020; Zeng et al., 2020). To expand our understanding about the role of m6A modification in tumorigenesis, provide valuable biomarkers for diagnostic and prognostic evaluation, and propose new therapeutic targets, it is necessary to gain a better understanding about the molecular and biological characteristics of m6A regulatory factors.

It was found in our study that the mutation rate of m6A regulators was relatively low in LUAD, ranging from 0 to 3%, among which the mutation rate of ZC3H13 was the highest (3%), and this mutation status did not affect the expression of other m6A regulators, indicating that the genetic distortion of m6A regulators may be more than the state change of a single gene. Through the study of the CNV characteristics of m6A regulators, we found that YTHDF1, FMR1, IGF2BP2, METTL3, HNRNPC, IGF2BP3, YTHDF3, IGF2BP1, HNRNPA2B1, LRPPRC, YTHDC1, and FTO had the advantage of increasing the CNV, while YTHDF2, WTAP, YTHDC2, RBM15, ZC3H13, and RBM15B had the advantage of reducing the CNV. Subsequent differential analysis revealed that these m6A regulators were differentially expressed among lung tumors and normal tissues. Relating gene CNV to RNA expression, we found that CNV-amplified m6A regulators (METTL3, YTHDF1, HNRNPC, LRPPRC, HNRNPA2B1, IGF2BP1, IGF2BP2, and IGF2BP3) were highly expressed in LUAD tissues as compared with those in normal lung tissues, and vice versa for FTO, suggesting that CNV change may be a significant factor leading to perturbations of m6A regulator expressions.

Based on the expression characteristics of m6A regulators, we identified three subtypes with different TME characteristics and prognosis. Our study found that patients with m6Acuster-A had a significant survival advantage, while m6Acluster-C subtype had the worst prognosis (Figure 2B). To further explore whether m6A regulators play a role in prognosis among different patients, we analyzed the gene expression patterns of m6A regulators among different m6Aclusters. The results revealed that the expression of these m6A regulators differed significantly in different m6A decoration patterns. The expression levels of prognostic risk genes (WTAP, RBM15, HNRNPC, LRPPRC, IGF2BP2, and IGF2BP3) were significantly increased in the m6Acluster-C subtype, while the prognostic-friendly genes (YTHDC1, YTHDC2, and FTO) were apparently increased in the m6Acluster-A subtype. This suggested that the perturbation of m6A regulator expression had a great impact on patient prognosis, thereby distinguishing m6A regulatory patterns with different characteristics. Geeleher et al. (2014a) found that TME structure played a key role in tumor progression and affected the immunotherapy effect. Baseline levels of tumor-infiltrating CD4+/CD8+ T cells, NK cells, M1 macrophage, and inflammatory cytokine secretion were demonstrated to be associated with immune response (Topalian et al., 2016; Galon and Bruni, 2019). In addition, PD-L1 and TMB are recognized as biomarkers for forecasting anti–PD-1/L1 treatment response. In this study, we found that antitumor lymphocyte subsets including activated CD4+/CD8+ T cells and NK T cells were mainly enriched in m6Acluster-B and m6Acluster-C subtypes, both of which exhibited higher PD-L1 expression, TMB, and poorer prognosis. This supported the potential predictive value of the three m6A-related subtypes in terms of immunotherapeutic response and prognosis. Furthermore, from DEGs identified in different m6A decoration patterns, we obtained three transcriptome subtypes according to the m6A signature gene and found that they were obviously correlated with various survival outcomes and the TME landscape. Based on the DEGs associated with prognosis, we established a scoring system called “m6Ascore signature”. The m6Ascore was correlated with TME characterization, immune cell infiltration, and also with predictors of immune response (expression of immune checkpoints) in LUAD, suggesting that m6A modification may affect the efficacy of immunotherapy. Tumors with infiltration of immune cells, especially CD8+ T cells, and high expression of PD-L1 in tumor cells and stroma were usually determined as thermo-tumors, and they were often more closely associated with immune checkpoint inhibitors (Chen and Mellman, 2017; Wu et al., 2018). It was found in our study that tumors with low m6Ascore had more aggregation of antitumor lymphocyte subsets, such as activated CD4+/CD8+ T cells and NK T cells. Moreover, this group of tumors had higher tumor mutation load and immune checkpoint expression. In addition, they also showed lower sensitivity to targeted therapies and poorer prognosis. This not only proved that m6Ascore signature had the potential to predict the curative effect of immune targeted therapy and prognosis of patients, but more importantly, it highlighted the importance of m6A modification in shaping tumor immunity. Interestingly, multivariate Cox analysis identified WTAP as an independent prognostic gene for LUAD patients, while this gene showed no statistical significance for OS in LUSC patients, suggesting that m6A regulators displayed different clinical value in various tumors (Gu et al., 2021).

Integrating different independent studies on common key characteristics of disease has become a preferred strategy. Since there may be deviations in a single experiment, it is necessary to seek the findings supported by several evidences to improve the reliability. Based on a larger LUAD cohort, this study disclosed the methylation modification pattern, TME landscape, and clinical significance of m6A regulatory factors in LUAD. Although we reviewed the literature and planned 23 recognized RNA methylation regulators, only 17 genes integrating multiple data sets were finally included. It is, therefore, necessary to incorporate more newly identified m6A regulators into the model to optimize the accuracy of m6A refitted decorative patterns. In addition, although a large number of retrospective data sets were used in this study to identify different m6A modification patterns and m6Ascore, there is still a lack of appropriate LUAD data sets based on immunotherapy regimens to verify the predictive robustness of m6Ascore to further strengthen our conclusions. Therefore, a prospective study involving a cohort of LUAD patients receiving immunotherapy is required to confirm our findings.

## Conclusion

Based on m6A regulators, the present study conducted an integrated assessment of m6A modification patterns in more than 1,900 LUAD samples to characterize the multidimensional profile of m6A regulators in LUAD. It was found that m6A regulators served an indispensable role in shaping heterogeneity and sophistication of the TME. According to these findings, we postulated that the evaluation of the m6A modification pattern of LUAD would help better understand the infiltration characteristics of the TME and provided important insights into the effectiveness of immunotherapy. m6A modification could be a promising strategy for the management of LUAD.

## Data Availability

The datasets presented in this study can be found in online repositories. The names of the repository/repositories and accession number(s) can be found in the article/Supplementary Material.

## Supplementary Material

Supplementary Figure S1 Effect of the mutation status of ZC3H13 on the expression of other m6A regulators in TCGA–LUAD cohort.

Supplementary Figure S2
**(A)** Univariate and **(B)** multivariate Cox regression analyses of 17 m6A regulatory factors. **(C)** Unsupervised clustering of the m6A regulators and consensus matrices for k = 3. **(D)** Unsupervised clustering of 17 m6A regulators. The m6Acluster, project, age, sex, smoking status, and living condition were used as patient notes. Red indicated the high expression of regulatory factors, while green represented the low expression. **(E)** Unsupervised clustering of the m6A phenotype–related genes correlated with prognosis and consensus matrices for k = 3. **(F)** GSVA enrichment analysis of the activation state of biological pathways in different m6A modification patterns (m6Acluster B vs. m6Acluster A). Heat maps of these biological processes, with red representing activated pathways and blue representing inhibited pathways, using LUAD queue as sample annotation. **(G)** Expression of 17 m6A regulatory factors in the three gene clusters. The upper and lower ends of the box represented the quartile range of values. The lines in the box represented intermediate values, and the real points represented outliers. The asterisk represented the statistical p value. (****p* < 0.001; ***p* < 0.01; **p* < 0.05). The statistical differences between the three gene clusters were tested by using one-way ANOVA.

Supplementary Figure S3 Predictive performance of m6Ascore in various subgroups of patients with LUAD. **(A)** Age ≤ 65 subset. **(B)** Age > 65 subset. **(C)** Female subset. **(D)** Male subset. **(E)** Ever smoking subset. **(F)** Never smoking subset. **(G)** Stage I/II subset **(H)** Stage III/IV stage. **(I)** EGFR wt subset. **(J)** STK11 wt subset. **(K)** Kras wt subset. **(L)** Kras mut subset. **(M)** TP53 wt subset. **(N)** TP53 mut subset.

Supplementary Figure S4 Relationship between m6Ascore and clinical parameters. **(A)** Age. **(B)** Sex. **(C)** Smoking status. **(D)** Stage. **(E)** EGFR. **(F)** STK11. **(G)** Kras. **(H)** TP53. **(I)** Survival status. The right side of each figure showed the proportion of high- and low-m6Ascore groups in each clinical characteristic, and the left side showed the comparison of m6Ascores in each clinical subgroup.

Supplementary Figure S5
**(A)** Comparison of the TME infiltration cells abundances in the high- and low-m6Ascore groups. **(B)** Comparison of immune scores in the high- and low-m6Ascore groups. **(C)** Comparison of tumor purity in the high- and low-m6Ascore groups. **(D)** GSEA analysis of the high- and low-m6Ascore groups. **(E,F)** GSVA analysis of the high- and low-m6Ascore groups. E, KEGG; F, GO.

Supplementary Figure S6 Construction and evaluation of the nomogram. **(A)** Univariate Cox analysis of m6Ascore, m6Acluster, and some clinical parameters (age, sex, smoking status, and stage). **(B)** Multivariate Cox analysis of m6Ascore, m6Acluster, and some clinical parameters (age, sex, smoking status, and stage). **(C)** Nomogram for predicting the 3- and 5-year survival of patients. **(D)** Calibration curves of 3 and 5 years.
