# Supplementary figures and images for: Comprehensive Analysis Revealed the Potential Implications of m6A Regulators in Lung Adenocarcinoma
Source: Front Mol Biosci. 2022 Mar 28;9:806780. doi: 10.3389/fmolb.2022.806780 (PMC8995862; doi:10.3389/fmolb.2022.806780)

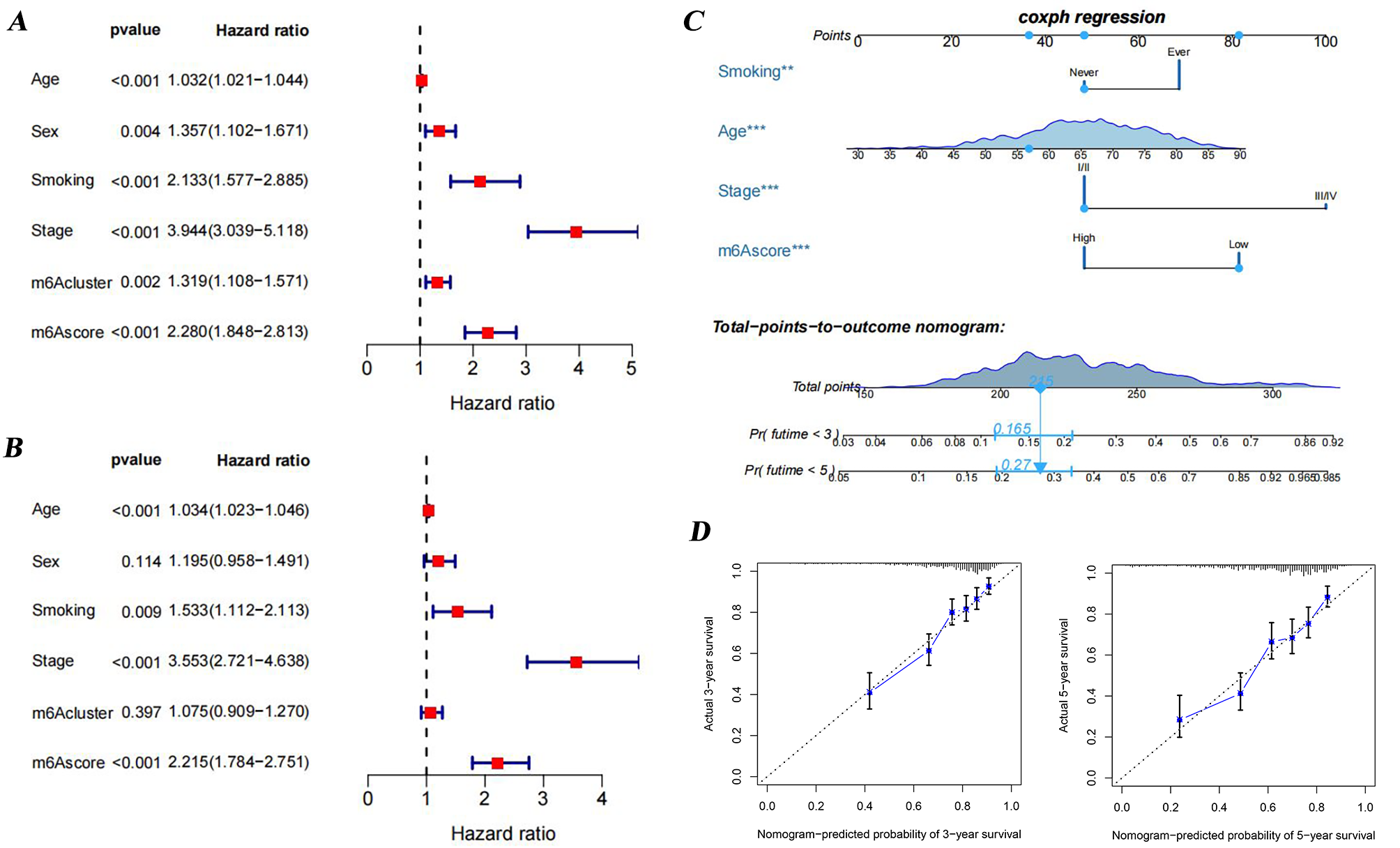

Supplement: Supplementary file 1 [file Image6.TIF]

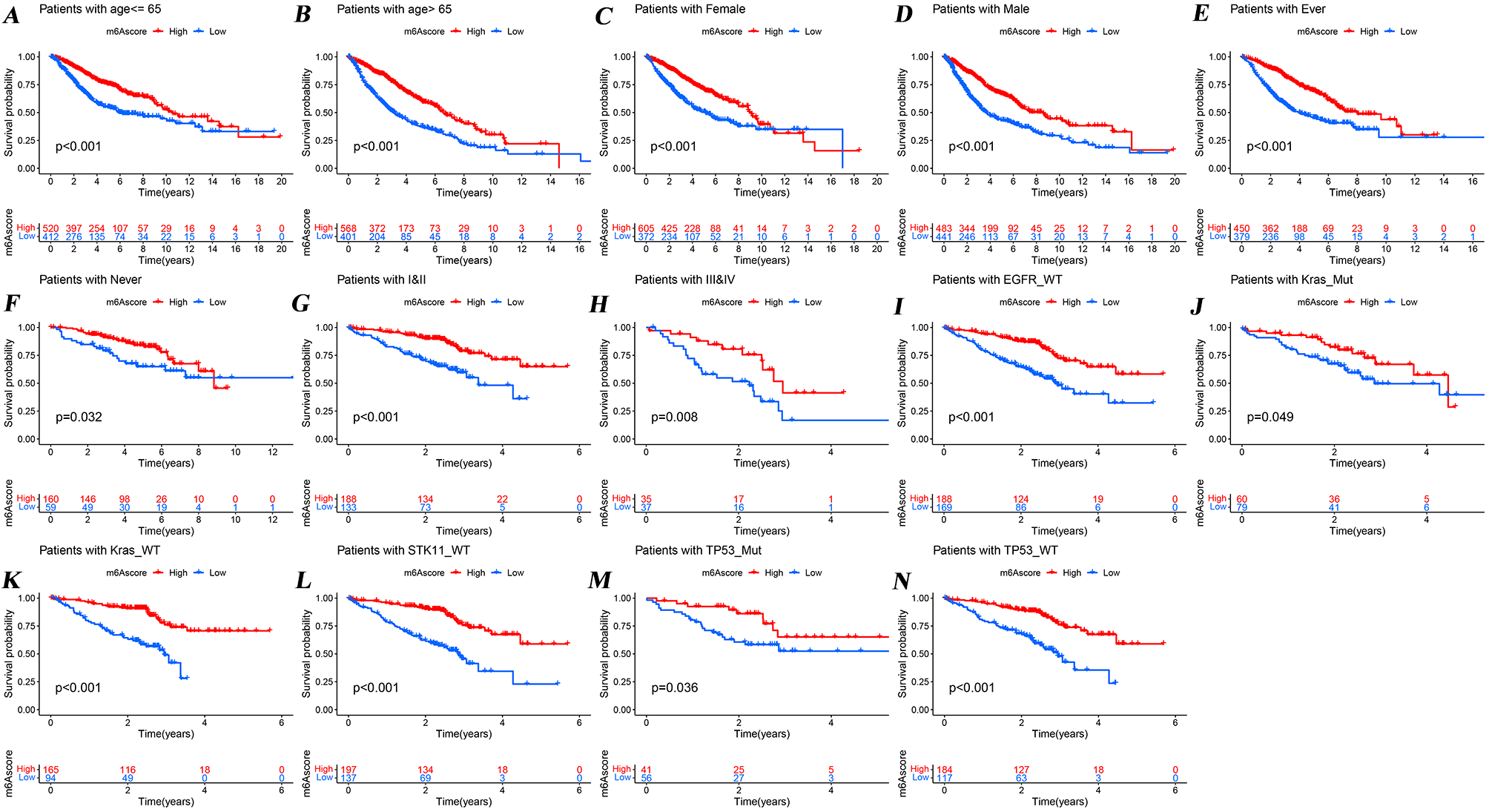

Supplement: Supplementary file 2 [file Image3.TIF]

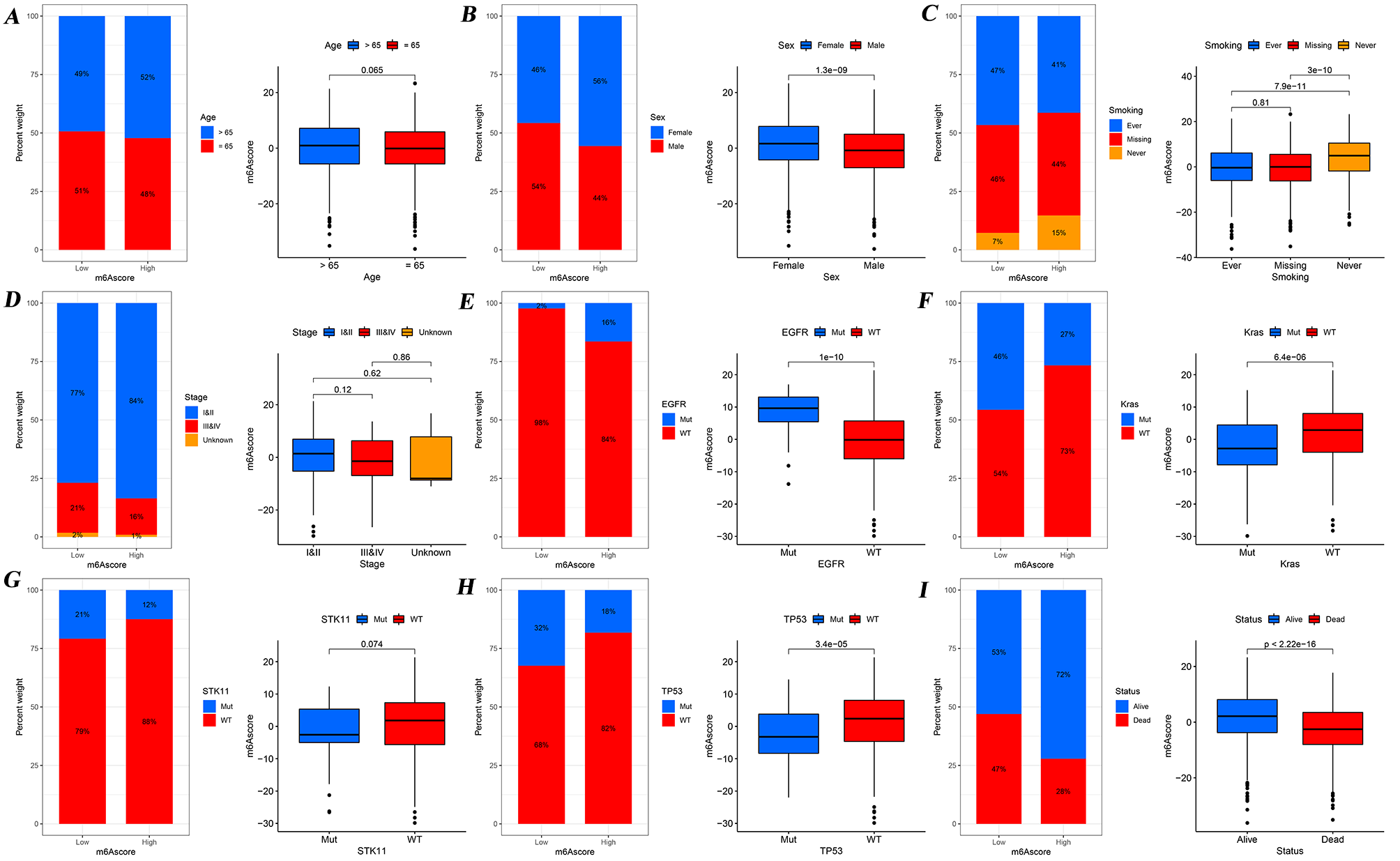

Supplement: Supplementary file 3 [file Image4.TIF]

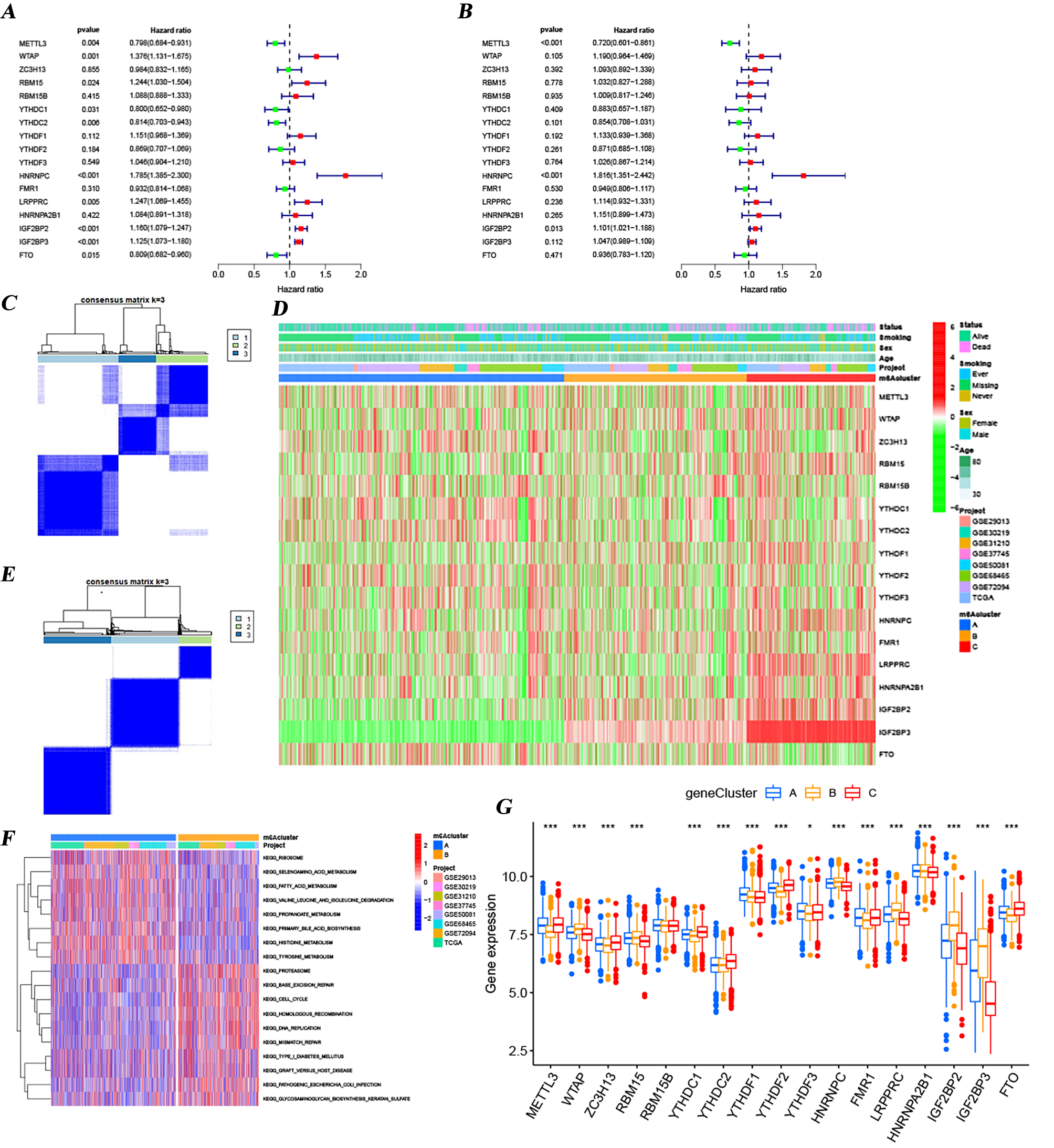

Supplement: Supplementary file 4 [file Image2.TIF]

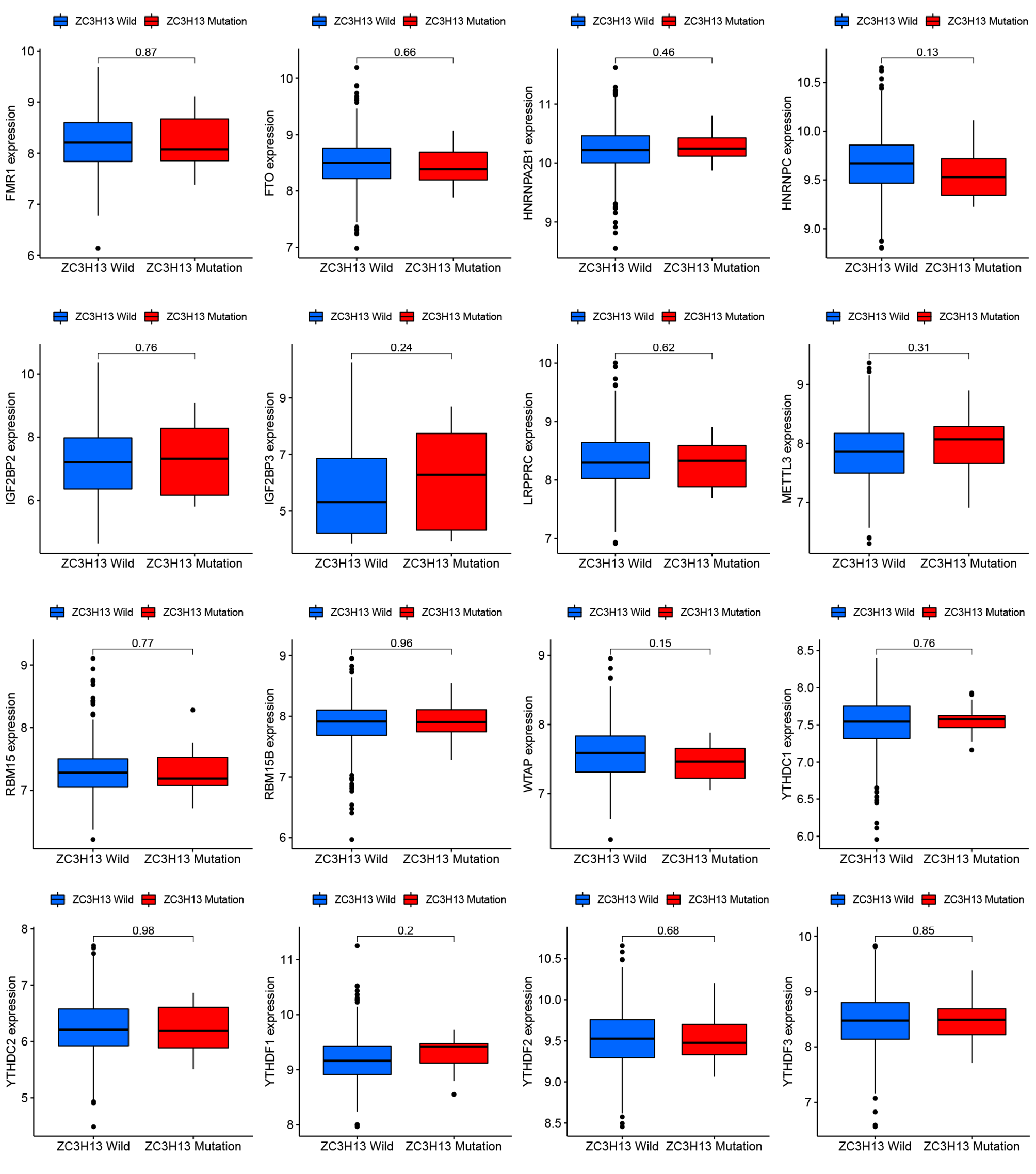

Supplement: Supplementary file 5 [file Image1.TIF]

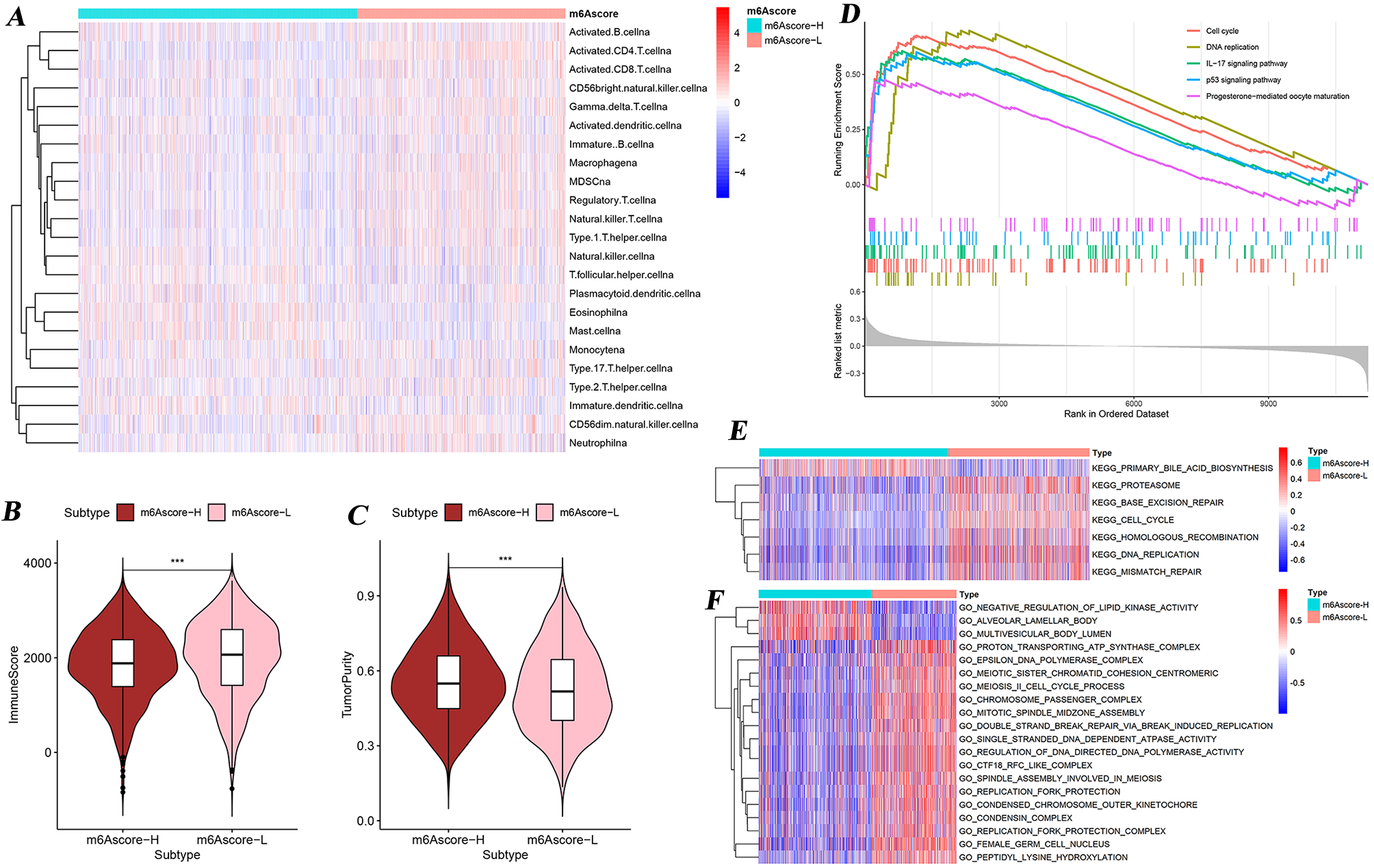

Supplement: Supplementary file 6 [file Image5.TIF]
